# Supplementary material for: SOX1 Functions as a Tumor Suppressor by Repressing HES1 in Lung Cancer
Source: Cancers (Basel). 2023 Apr 8;15(8):2207. doi: 10.3390/cancers15082207 (PMC10136456; doi:10.3390/cancers15082207)

Figure S1E
Full blot

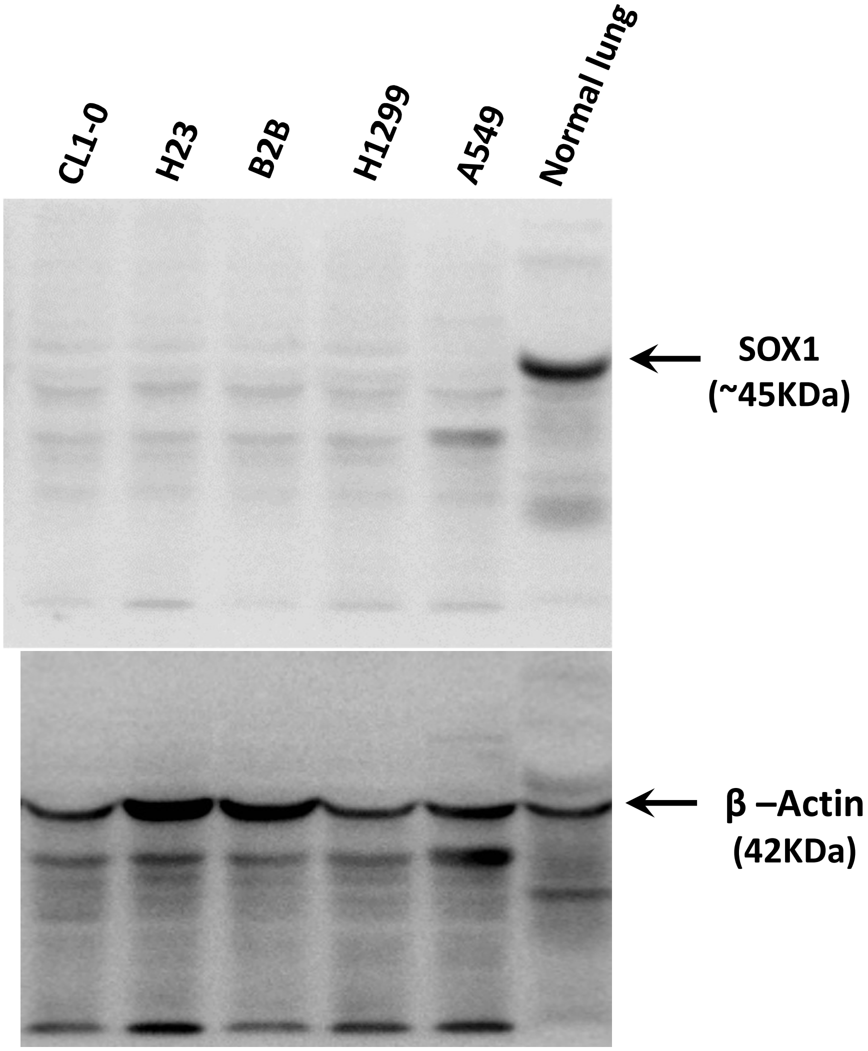

Replicate
Figure 1E Full blot

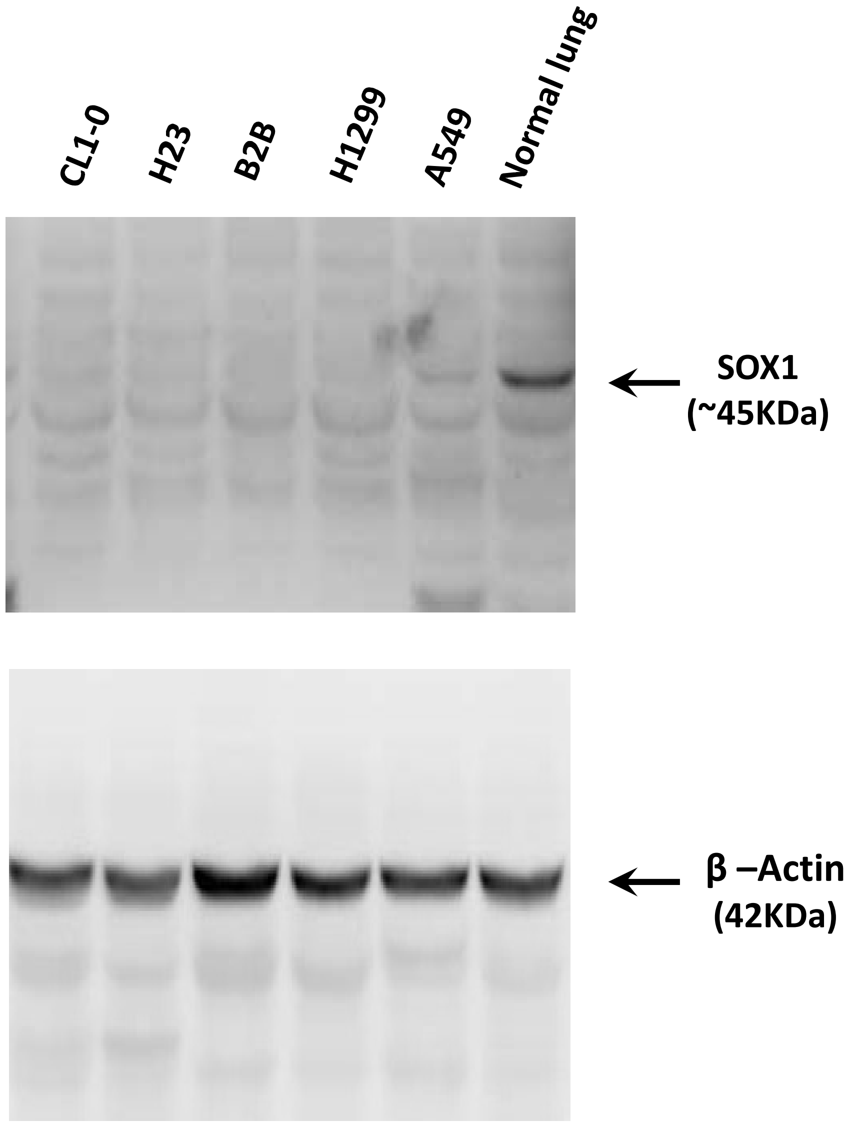

Figure 2 western blot full blot

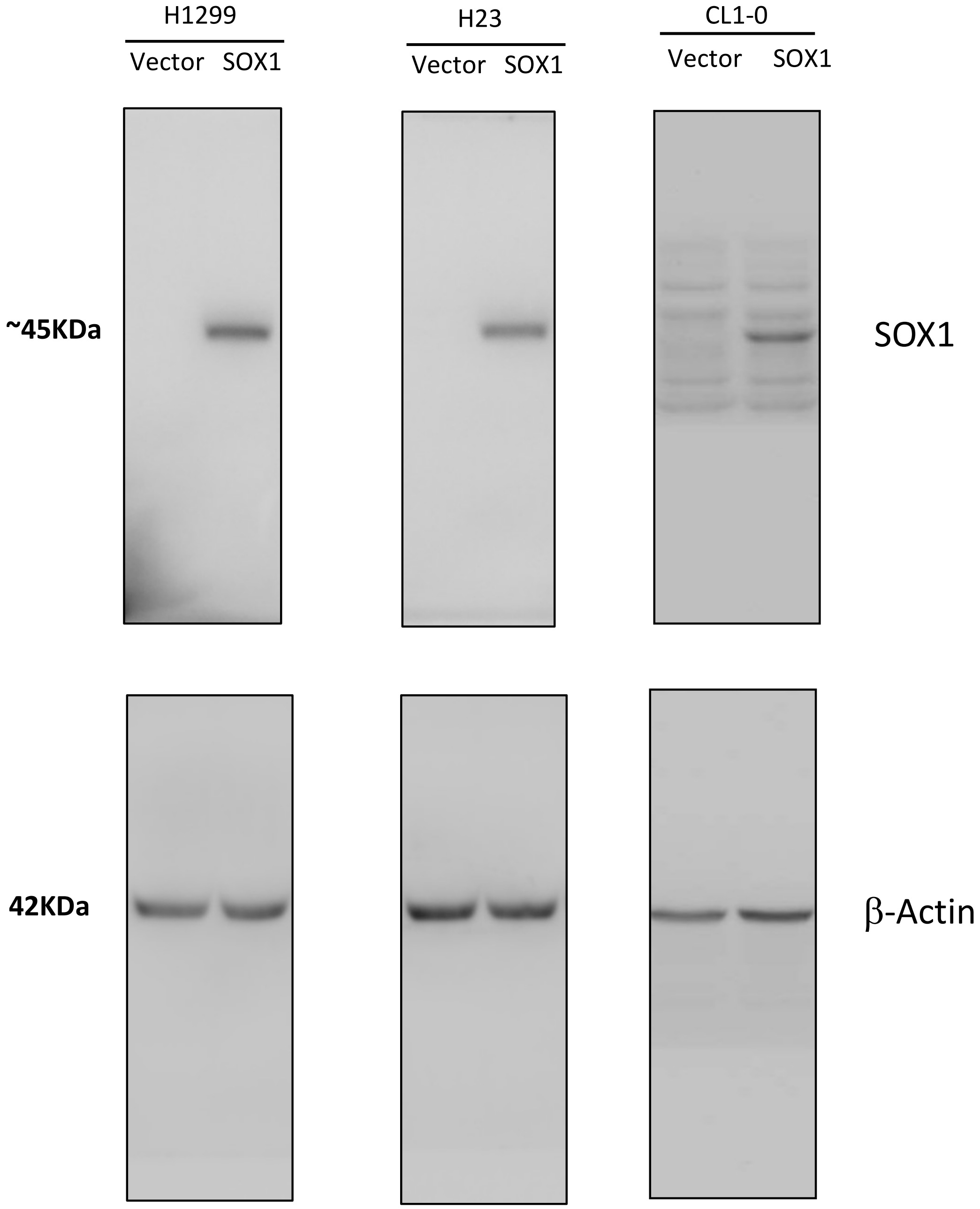

Replicate

Figure 2 western blot full blot

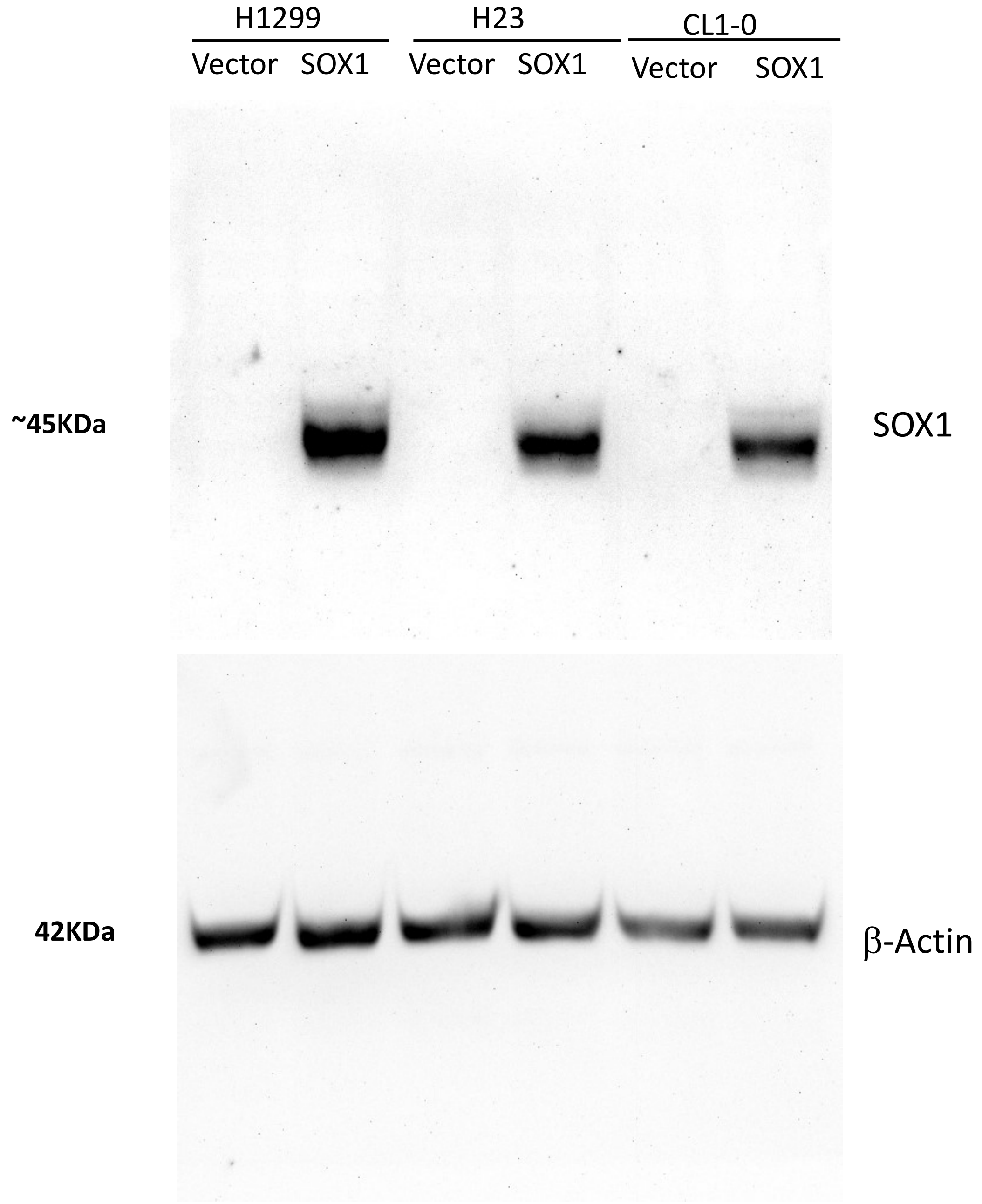

Figure 3 full blot (tissues, only one western blot)

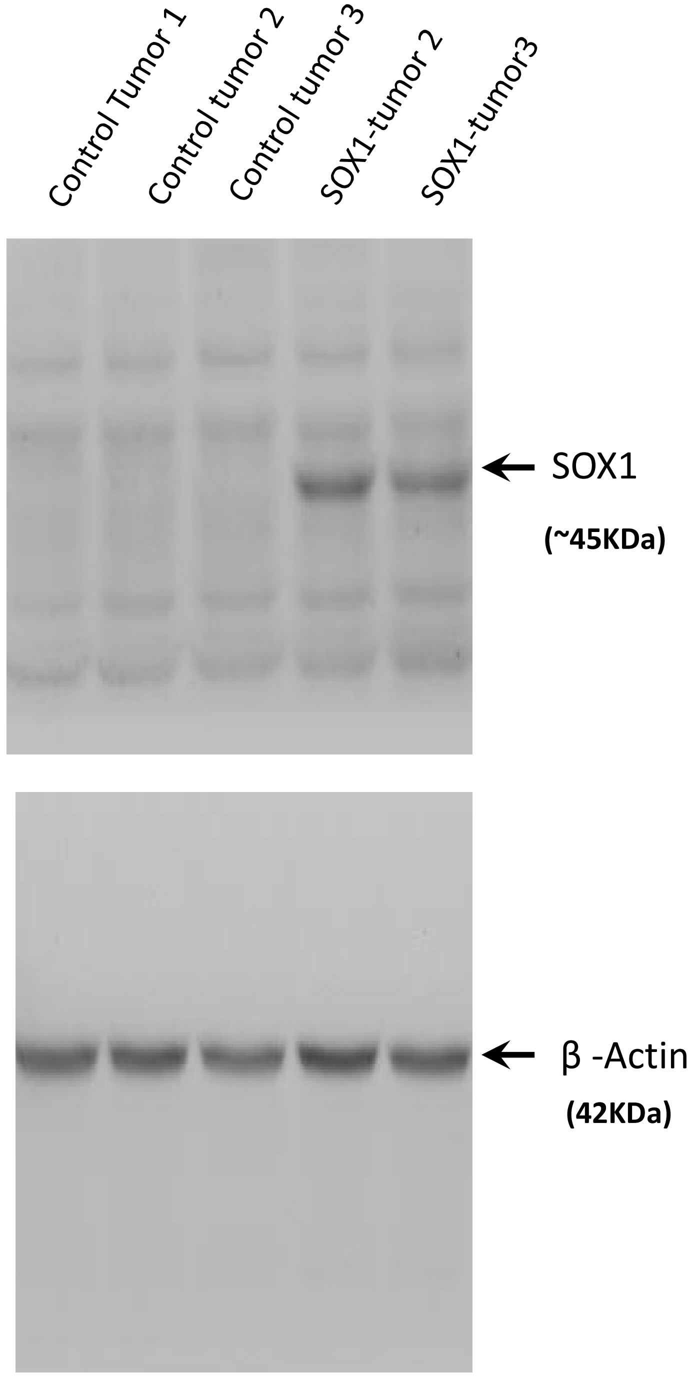

Figure 4 full blot

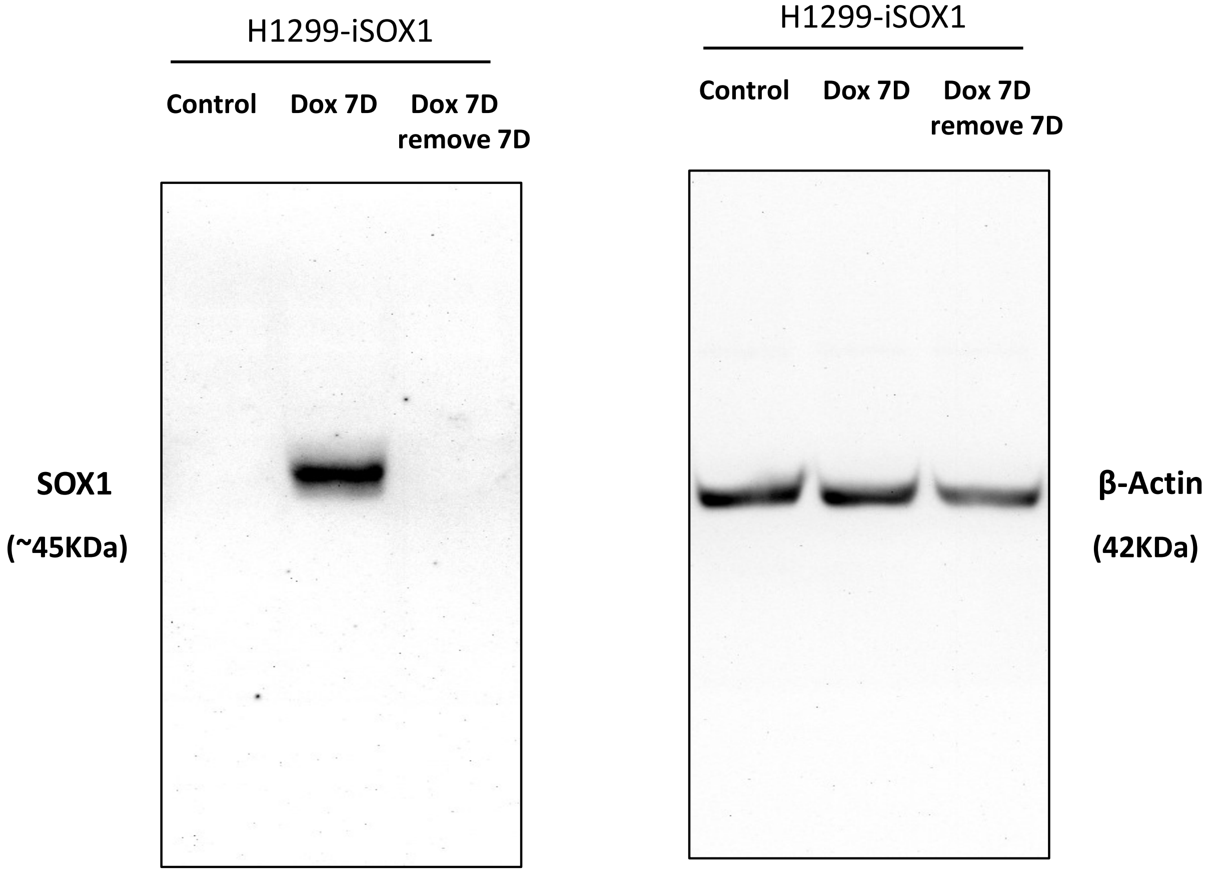

Figure 4 full blot (replicate)

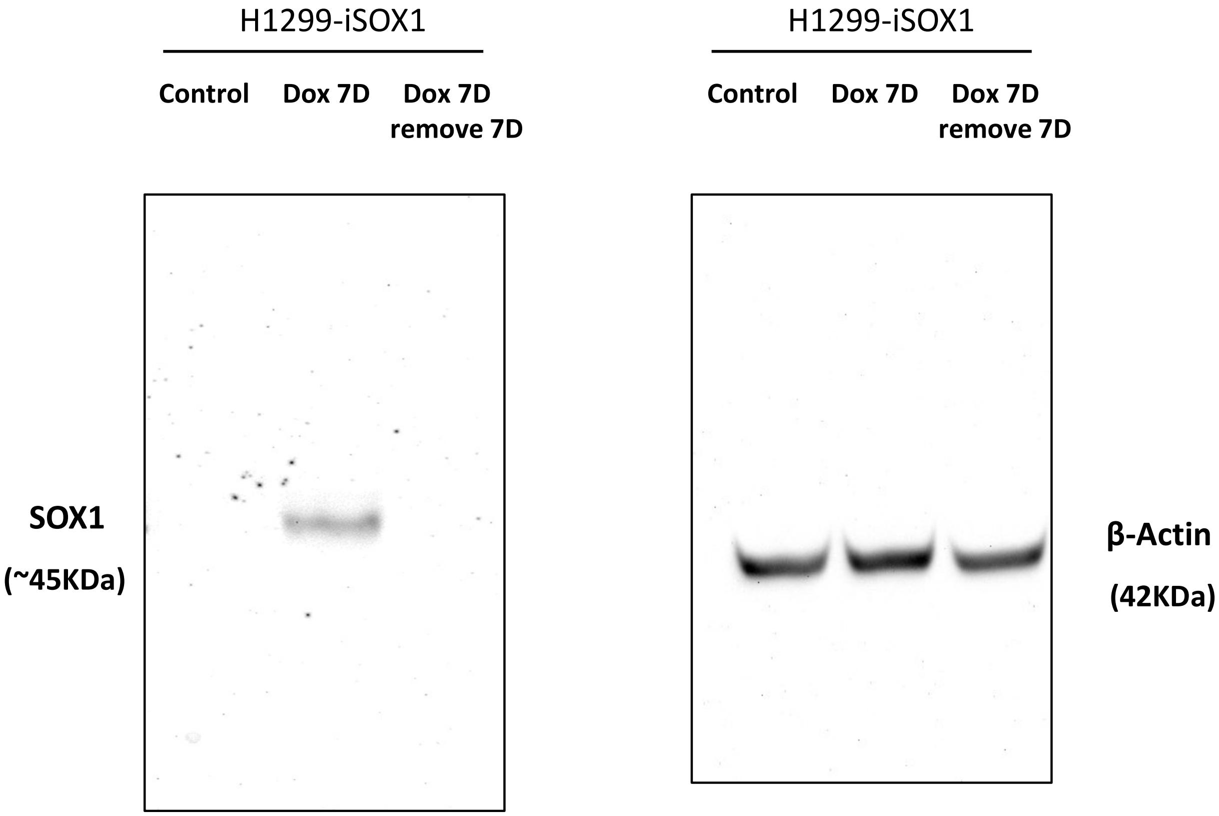

Figure 5 full blot

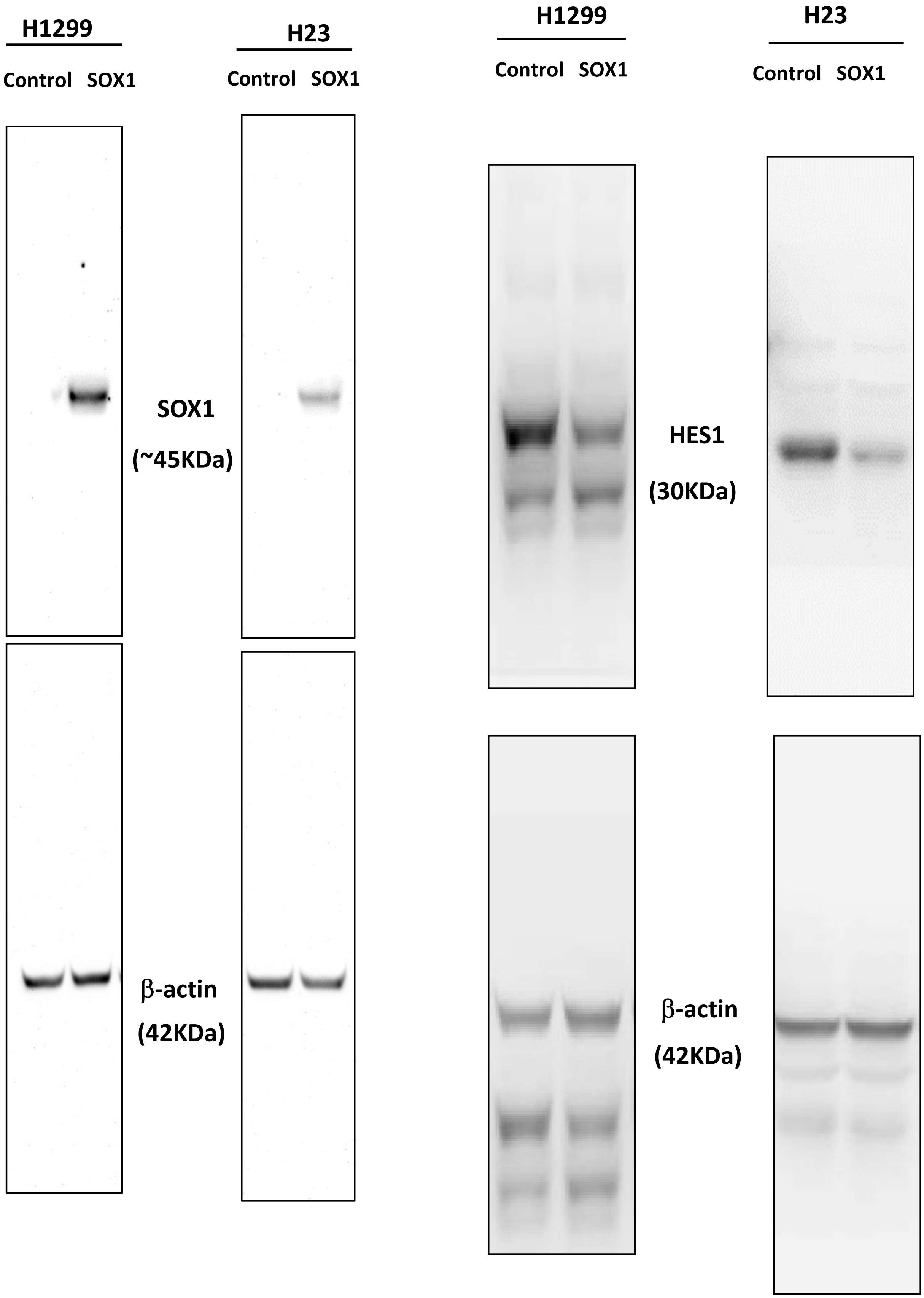

Figure 5 full blot replicate

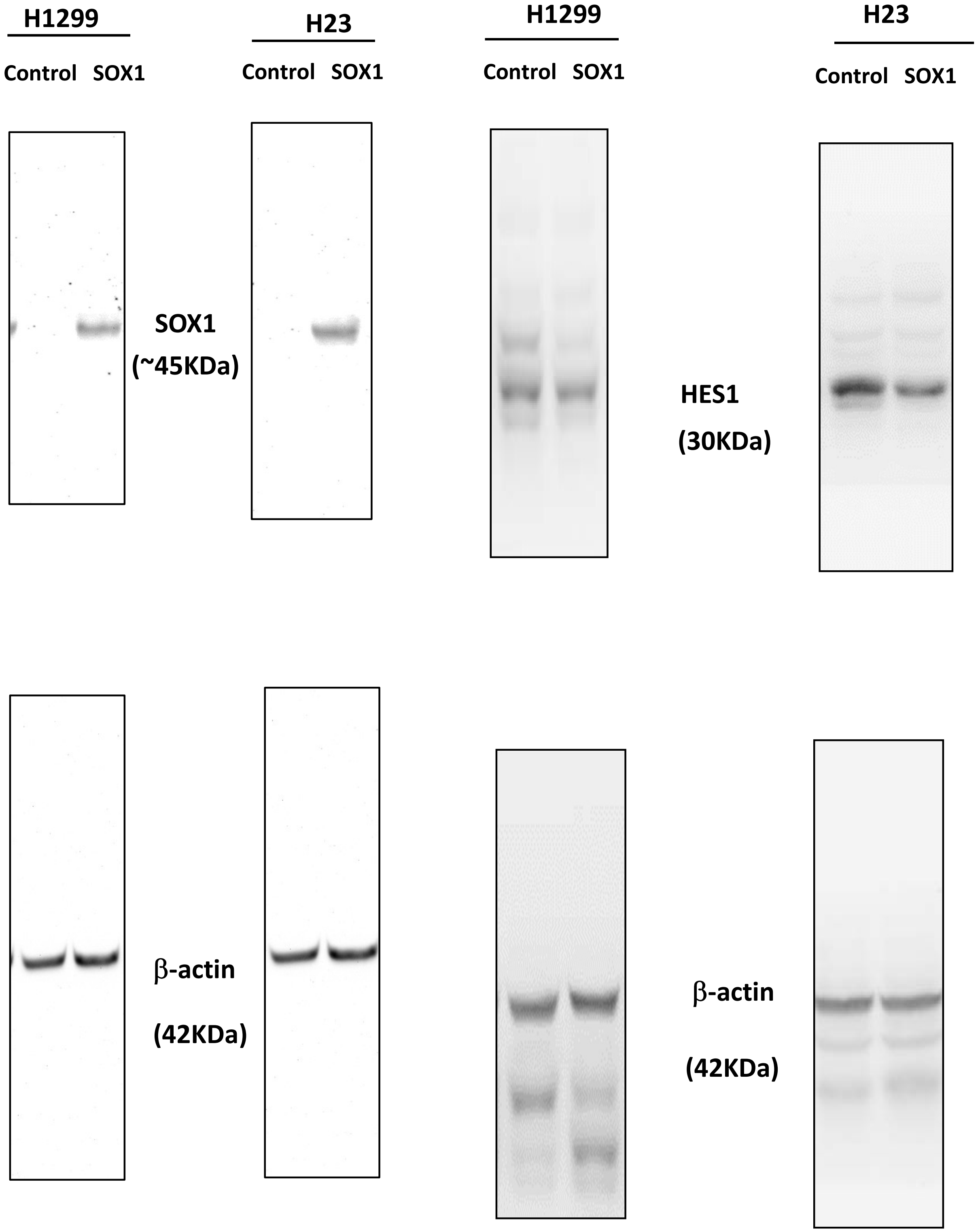

Figure 6 western blot full blot

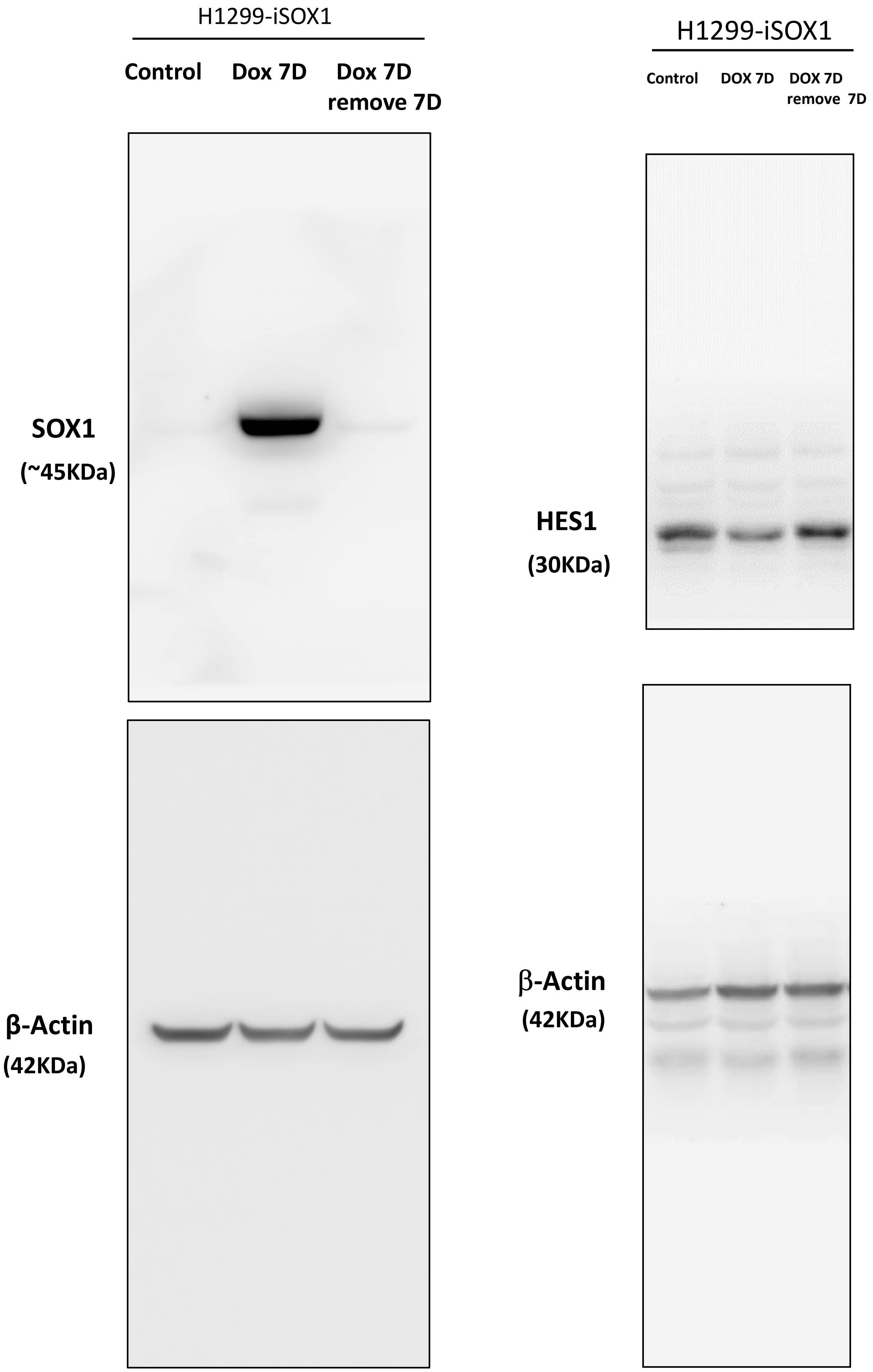

Figure 6 western blot full blot replicate

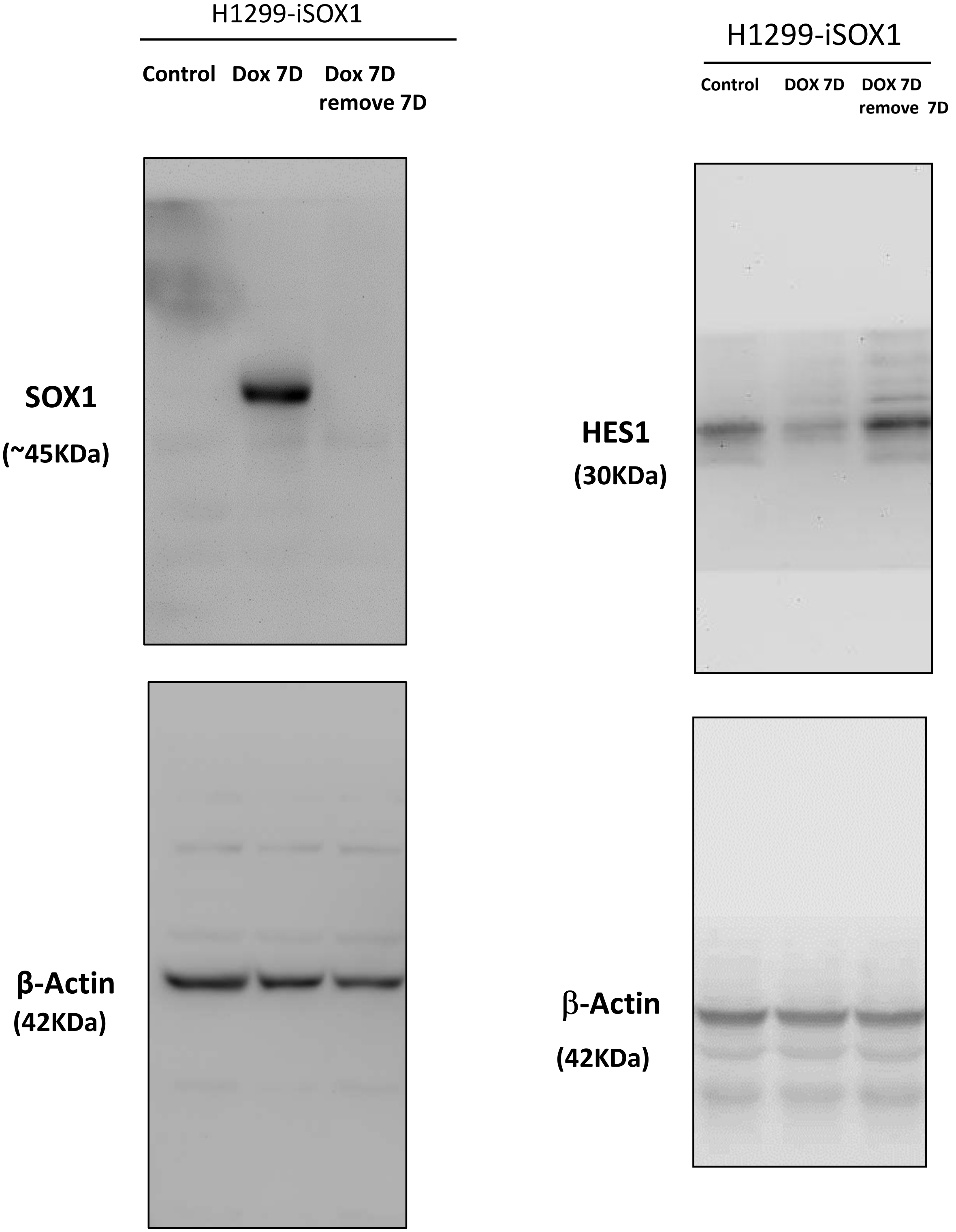

Figure 7 full blot

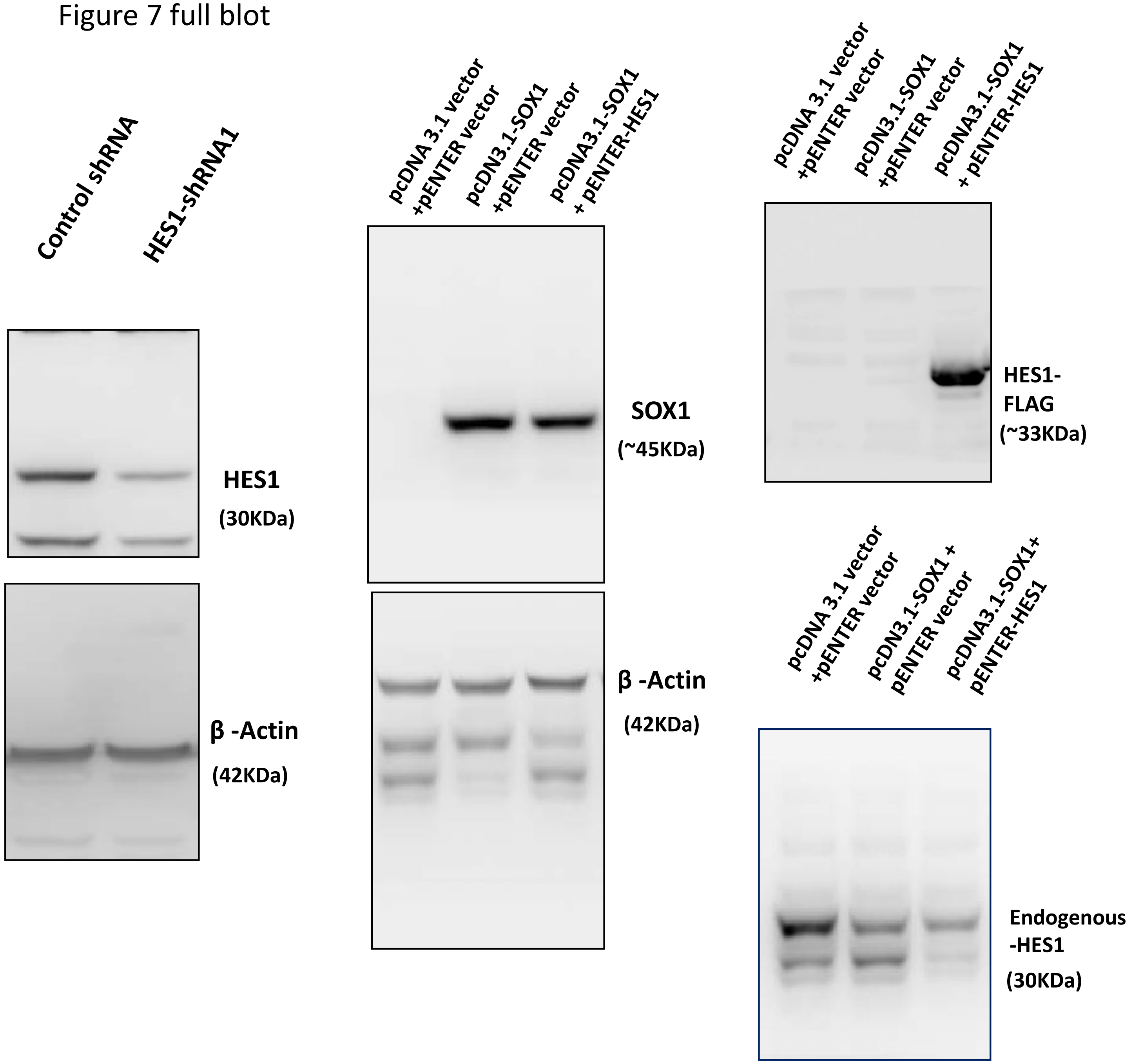

Figure 7 full blot replicate

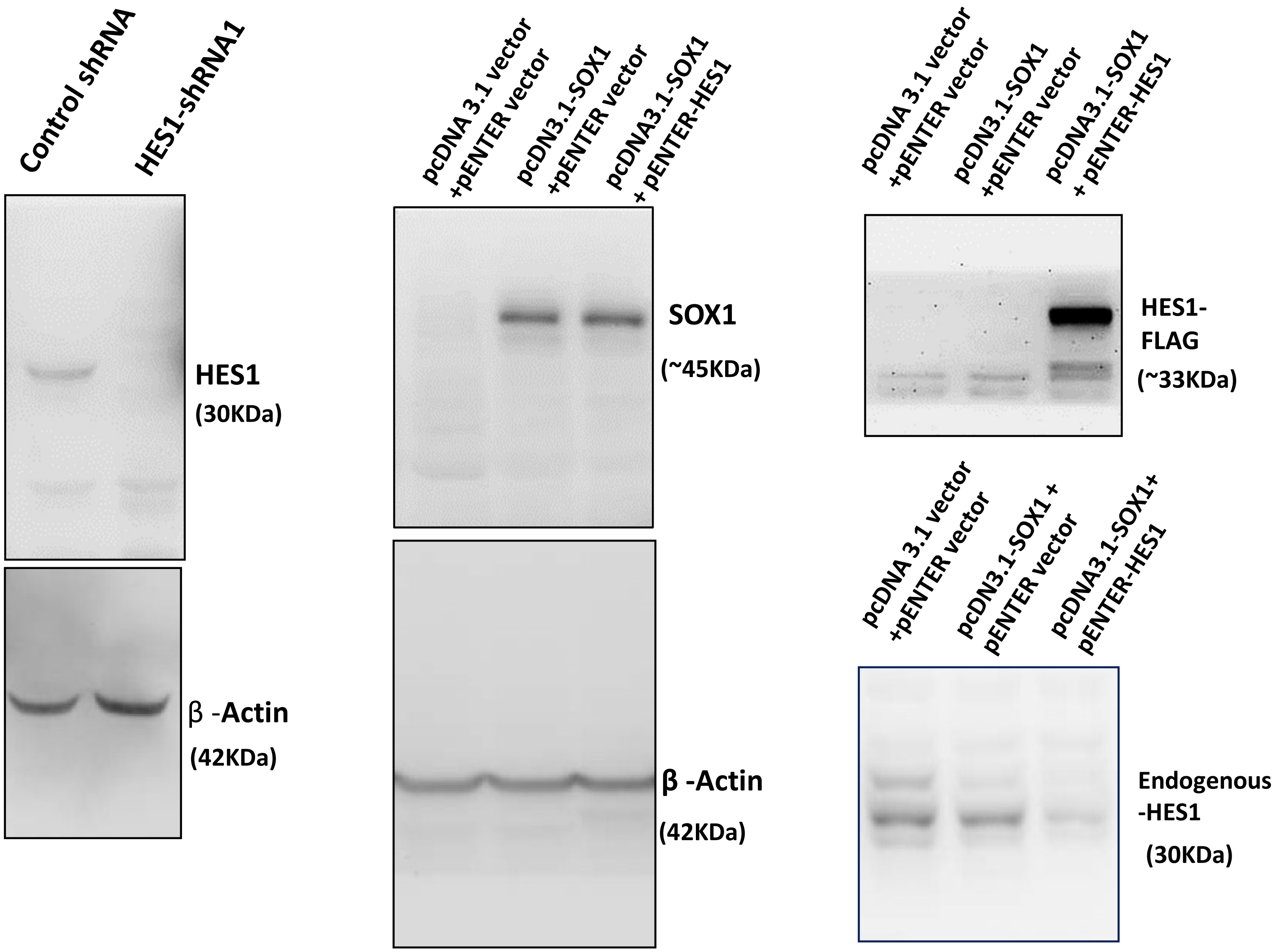

Supplement: Supplementary file 1 [file cancers-15-02207-s001.zip › Supplementary File S1.pdf]
