# Supplementary material for: SOX1 Functions as a Tumor Suppressor by Repressing HES1 in Lung Cancer
Source: Cancers (Basel). 2023 Apr 8;15(8):2207. doi: 10.3390/cancers15082207 (PMC10136456; doi:10.3390/cancers15082207)
Supplement: Supplementary file 1 [file cancers-15-02207-s001.zip › Supplementary information.pdf]

Supplementary Materials

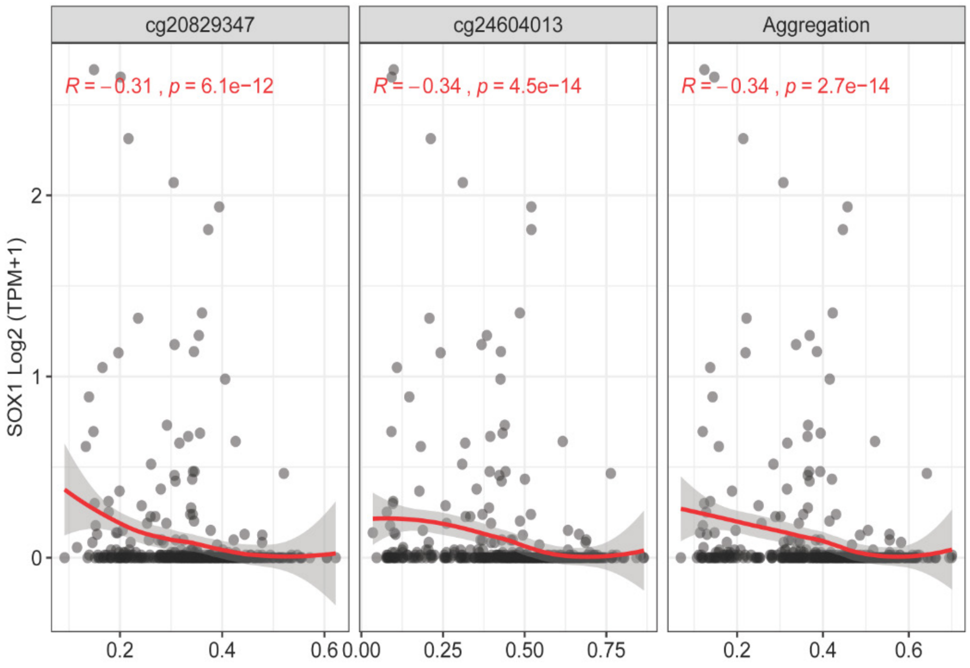

Figure S1. Correlation between SOX1 methylation and gene expression. We used the Shiny Methylation Analysis Resource Tool (SMART App (http://www.bioinfo-zs.com/smartapp) to analyze the dataset from TCGA. We performed pairwise correlation analysis to explore the correlation between the expression and DNA methylation using the Spearman method. Two CpG sites, cg20829347 and cg24604013, located in the promoter region of SOX1 are inversely related to SOX1 mRNA expression. DNA methylation beta value was the methylation index outputted for each probe site, which is ranging between 0 and 1, representing the ratio of the intensity of methylated signal to the intensity of total signal. SMART uses the log2-scaled (TPM+1) value (gene) and beta-value for calculation. Transcripts per million (TPM): mRNA transcript. Beta-value: methylation level. Aggregation: calculate the mean methylation for all the individual CpGs that users selected.

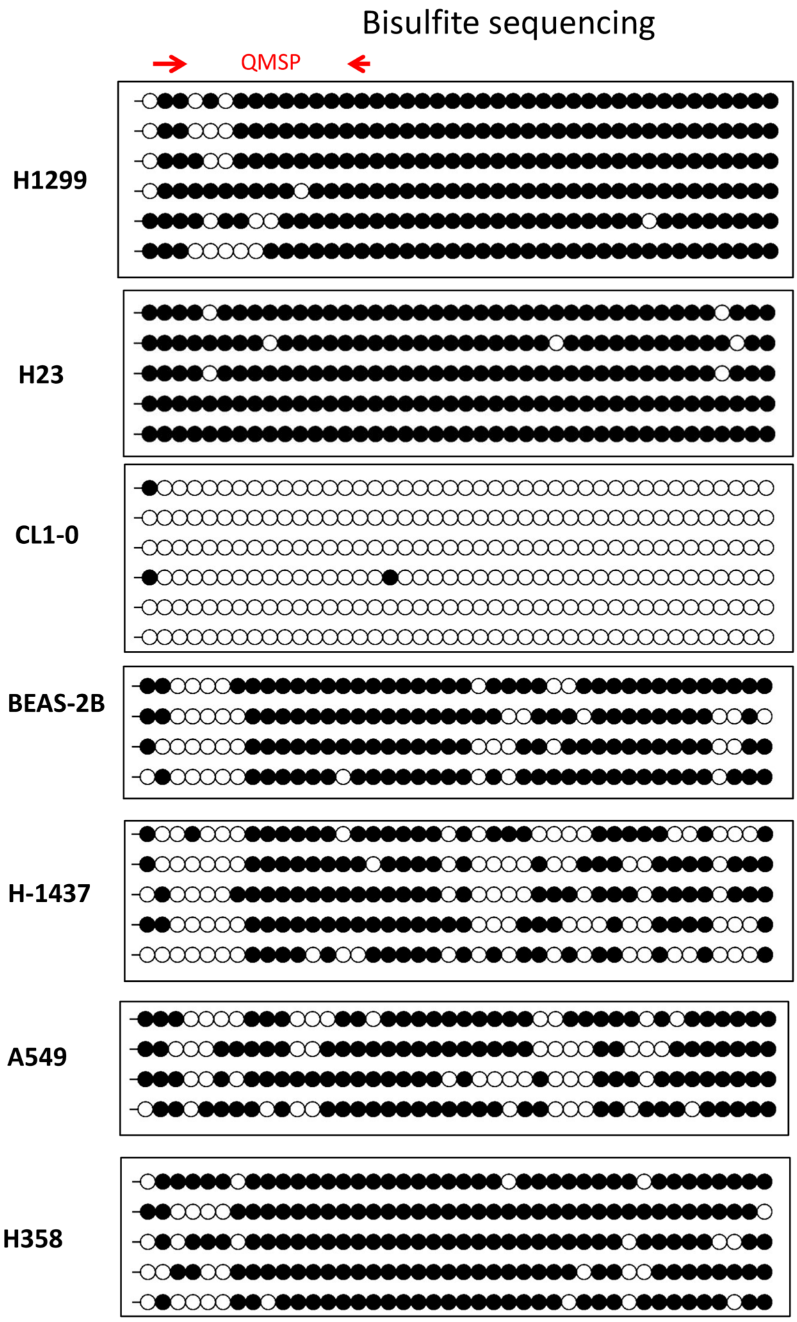

Figure S2. Bisulfite genomic sequencing analysis of the CpG sites located in the promoter region of SOX1. The SOX1 methylation status in 7 cell lines was analyzed by bisulfite genomic sequencing. Each clone is represented by a row, and 42 CpG sites are represented as circles. Black circles and white circles represent methylated and unmethylated CpG sites, respectively. Arrows indicate the locations of the Q-MSP primers. BS: Bisulfate sequencing; Q-MSP: quantitative methylation-specific PCR.

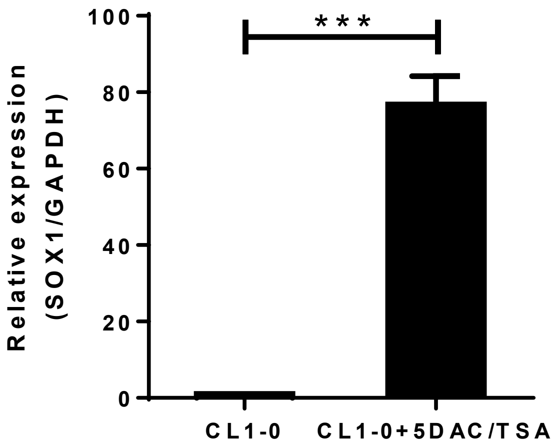

Figure S3. SOX1 was re-expressed after CL1-0 cells were treated with 0.5 μM DAC and 100 nM trichostatin A (TSA). Quantification of the gene expression levels of SOX1 and the internal reference GAPDH in CL1-0 cells treated with 0.5 μM 5DAC and 100 nM trichostatin A (TSA) or untreated were analyzed by quantitative RT–PCR. *** $p < 0.001$ (Student's t test).

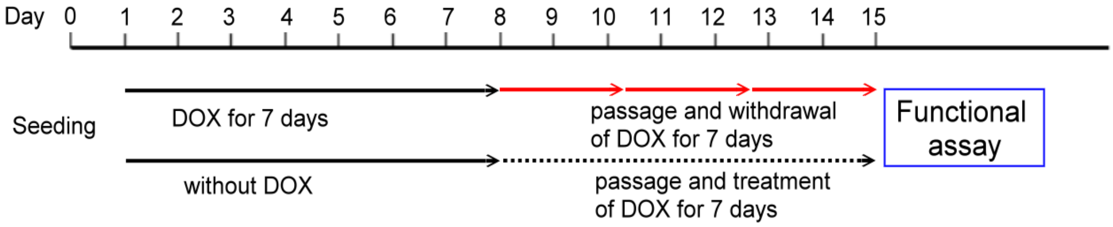

Figure S4. Illustration of SOX overexpression by the doxycycline (DOX)-inducible system. The detailed manipulation of SOX1 expression was illustrated, and MTS and AIG assays were performed according to the determined schedule.

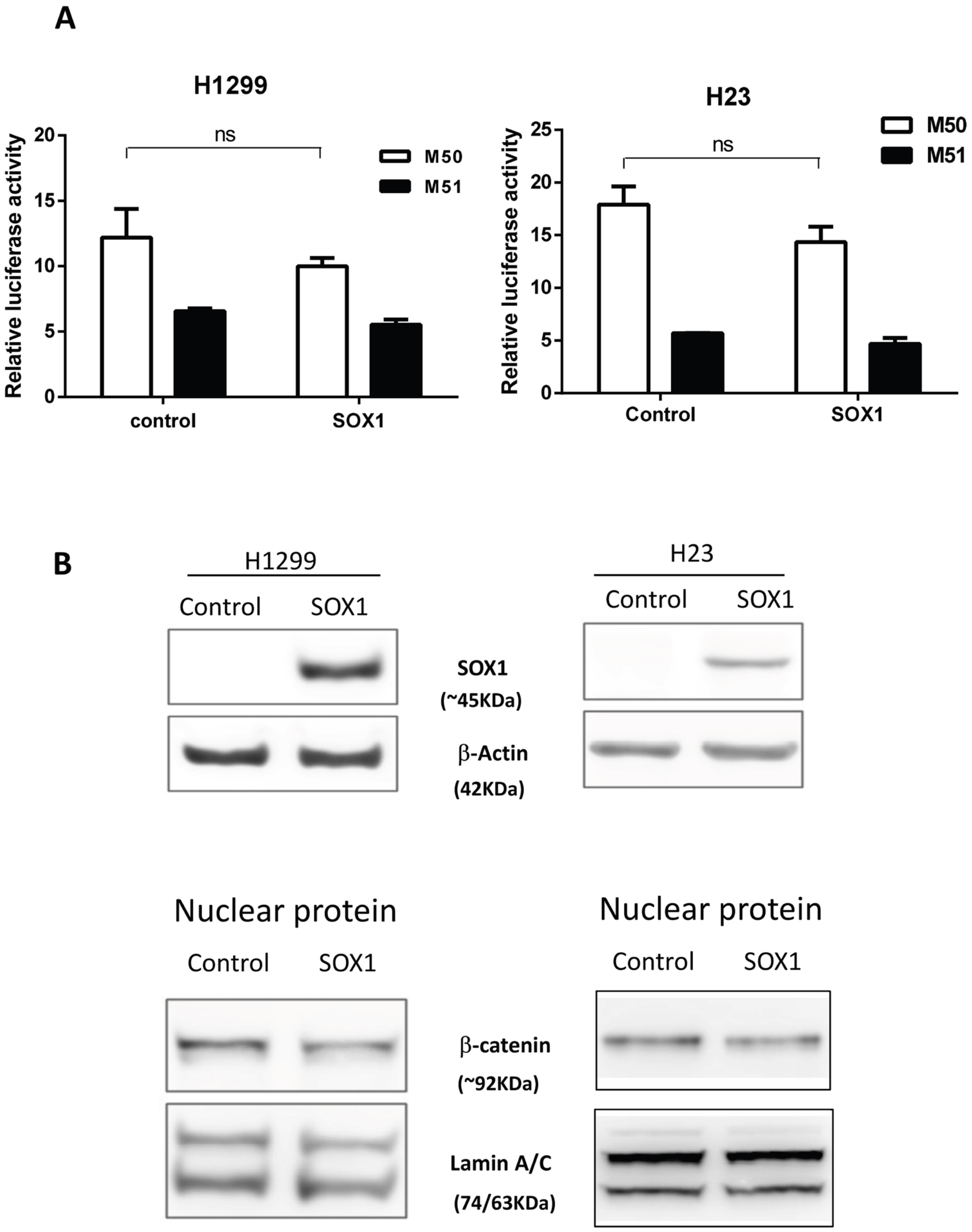

Figure S5. SOX1 did not significantly suppress Wnt/β-catenin-mediated TCF/LEF signaling in lung cancer cells. (A) A TCF luciferase reporter assay was performed using phRL-TK, TOPFLASH (the wild-type TCF reporter), or FOPFLASH (the mutant TCF reporter). The luciferase activity was normalized to the Renilla luciferase activity. The results are presented as the mean ± SE. The experiments were performed in triplicate. Significant differences were determined using the Mann–Whitney U test. ns: not significant. (B) Nuclear expression levels of β-catenin in lung cancer cells (H1299 and H23) were determined by western blotting. β-Actin and Lamin A/C were used as internal controls.

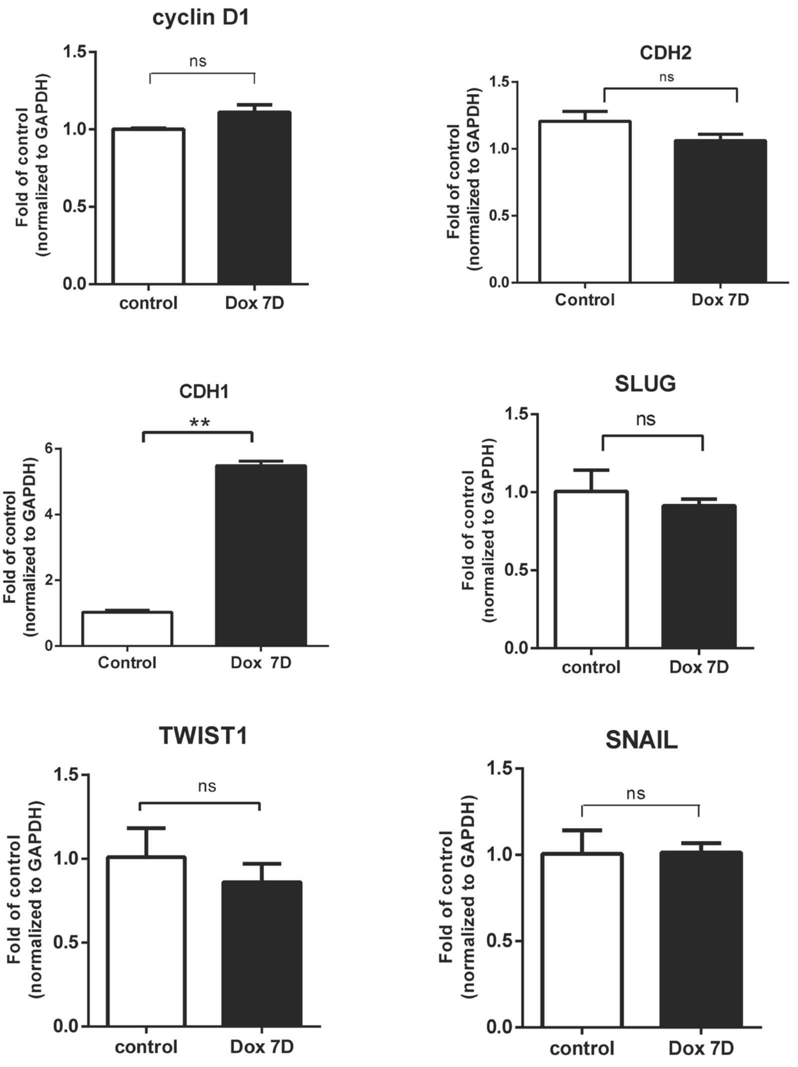

Figure S6. SOX1 did not significantly modulate the expression of molecules involved in Wnt/β-catenin and EMT in lung cancer cells with inducible SOX1 expression. The mRNA levels of cyclin D1, CDH1, CDH2, SLUG, TWIST1 and SNAIL in H1299 cells after treatment with DOX for 7 days were measured via quantitative RT–PCR. The data are shown as fold changes in mRNA expression relative to cells with control or doxycycline-inducible SOX1 transfectants. The data are expressed as the mean ± SE from three independent experiments. Significant differences were determined using the Mann–Whitney U test. **P < 0.01. ns: not significant.

A

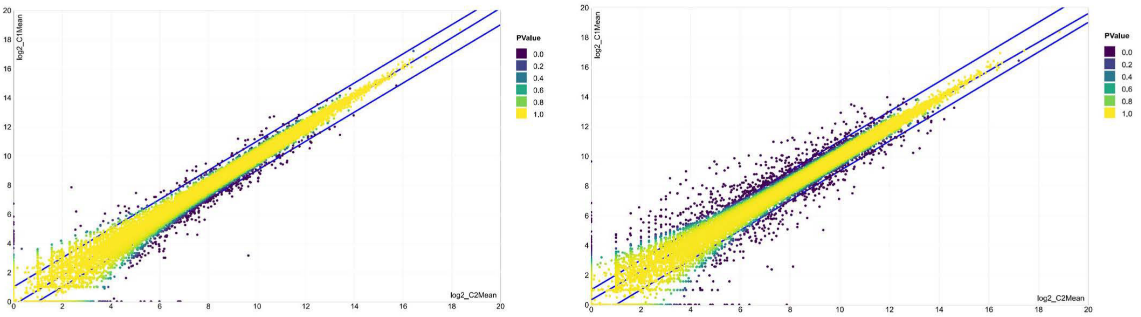

-Dox/+Dox

control/ +Dox

B

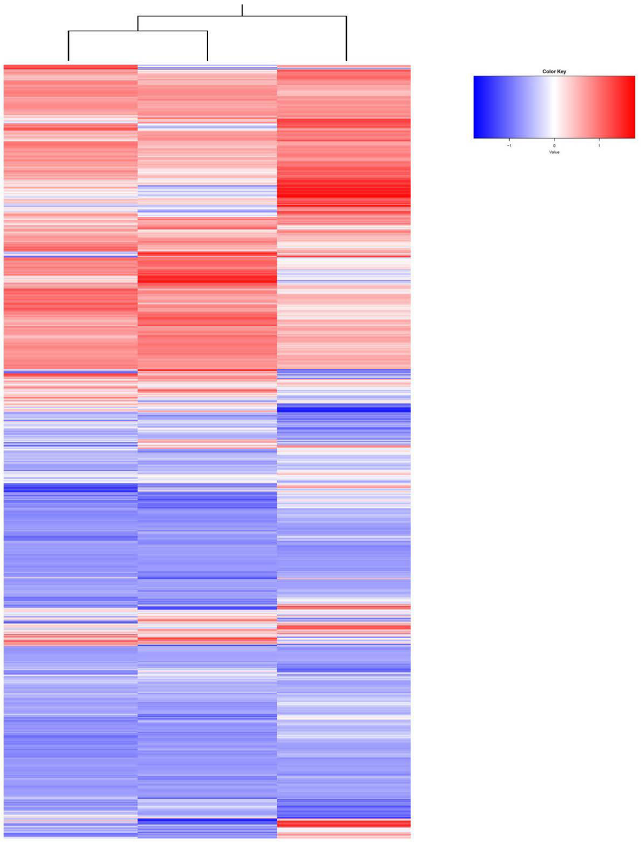

-Dox control +Dox

Figure S7. Scatter plot and heatmap showing the changes in gene expression after SOX1 induction and depletion in H1299 cells.

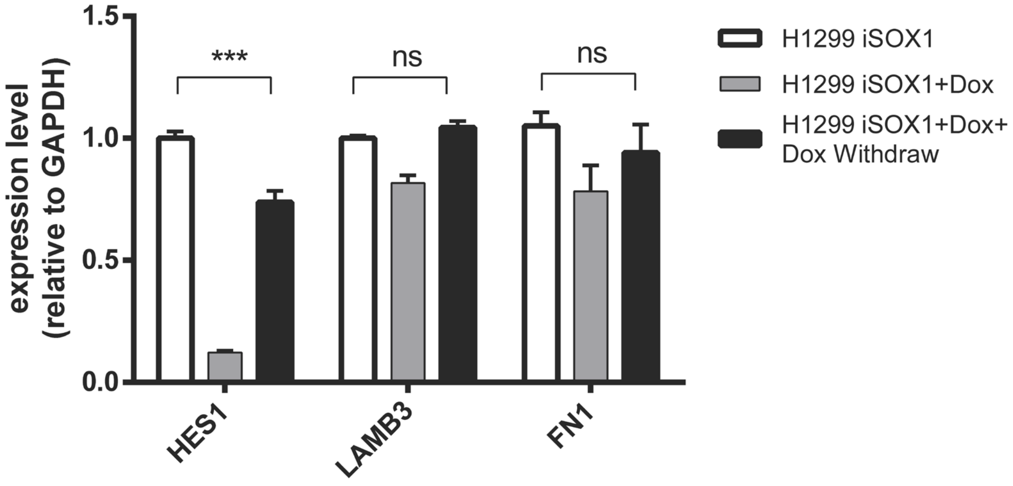

Figure S8. HES1 was repressed in inducible SOX1-expressing H1299 lung cancer cells. The mRNA levels of HES1, LAMB3 and FN1 in H1299 cells after treatment with DOX (1 μg/mL) or DOX treatment withdrawal were measured via quantitative RT–PCR. The data are shown as fold changes in mRNA expression relative to cells with control or doxycycline-inducible SOX1 expression. The data are expressed as the mean ± SE from three independent experiments. Significant differences were determined using the Mann–Whitney U test. ***P < 0.001. ns: not significant.

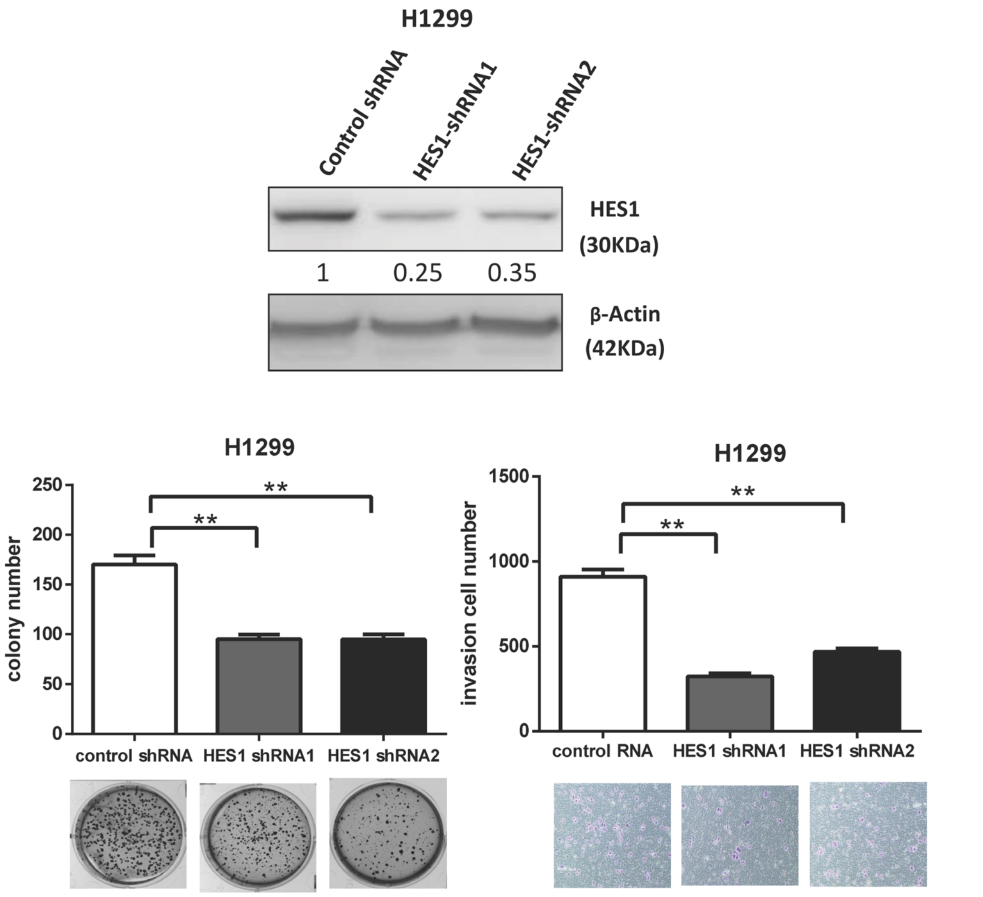

Figure S9. Knockdown of HES1 suppresses colony formation and cancer invasion in H1299 cells. The expression of SOX1 in H1299 cells transfected with control shRNA (shCtrl) or HES1 shRNA (HES1 shRNA1 and HES1 shRNA2) was analyzed by western blotting analysis. β-Actin was used as an internal control. The numbers in the western blots indicate the ratios of HES1 expression to that of the internal control. Colony formation and Matrigel invasion assays were used to analyze the effects on anchorage-independent growth and cancer invasion. The data are presented as the mean ± SE. Statistical significance was calculated with the Mann–Whitney U test. **P<0.01.

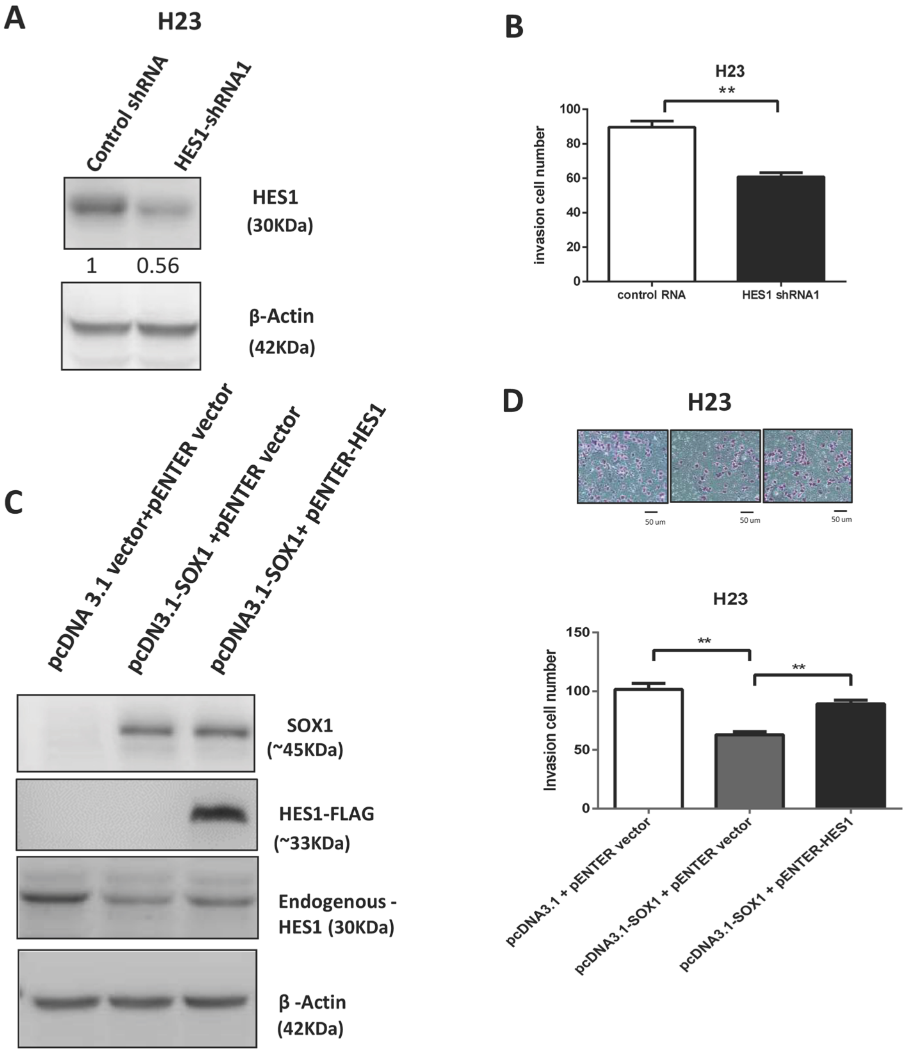

Figure S10. SOX1 represses cancer invasion by repressing HES1 in H23 lung cancer cells. (A) The expression of SOX1 in H23 cells transfected with control shRNA (shCtrl) or HES1 shRNA was determined by western blotting analysis. The numbers in the western blots show the ratios of HES1 expression to that of the internal control. (B) Invasion assays was performed in H23 cells. (C) H23 cells were transfected with the specific combination of vectors, and SOX1, HES1, and HES1-FLAG were analyzed by western blotting analysis. (D) Matrigel invasion assays were utilized to analyze the effects on cancer invasion. The data are presented as the mean ± SE. Statistical significance was determined with the Mann–Whitney U test. **P<0.01.

Table S1. Sequence of the oligonucleotides for making the shRNA construct-makings and quantitative PCR

qPCR primer	sense	antisense
SOX1	GAGATTCATCTCAGGATTGAGATTCTA	GGCCTACTGTAATCTTTTCTCCACT
CDH1	CCCACCACGTACAAGGGTC	ATGCCATCGTTGTTCACTGGA
CDH2	CAGATAGCCCGGTTTCATTTGA	CAGGCTTTGATCCCTCAGGAA
SNAIL	AATCGGAAGCCTAACTACAGCG	GTCCCAGATGAGCATTGGCA
SLUG	AAGCATTTCAACGCCTCCAAA	AGGATCTCTGGTTGTGGTATGAC
Cyclin D1	GAACAAACAGATCATCCGCAAAC	GCGGTAGTAGGACAGGAAGTTG
TWIST	AGCTACGCCTTCTCGGTCT	TCCTTCTCTGGAAACAATGACA
c-Myc	GGCAAAAGGTCAGAGTCTGG	GTGCATTTTCGGTTGTTGC
shRNA assay		
HES1 shRNA 1	GAAAGTCATCAAAGCCTATTA	
HES1 sRNA2	CCACGTGCGAGGGCGTTAATA	

PCR: polymerase chain reaction

**Table S2.** 53 Differential Expressed Genes(DEG)			
Gene	RPKM (H1299-SOX-DOX7)	RPKM (H1299-SOX-DOX7-Withdraw7)	RPKM (H1299-SOX-ori)
NBL1	0	56	60.7
POC1B-GALNT4	0	38.34	40.21
SMIM11A	0	28.13	28.39
CHKB-CPT1B	0	27.1	28.35
RBM14-RBM4	0	20.59	26.64
ADORA2A	4.24	228.96	151.96
ZNF664-FAM101A	0	17.83	19.2
RGPD6	0	17.38	17.04
FAM24B-CUZD1	0	17.37	14.42
NSFP1	0	16.07	13.97
SAA1	5	71.39	276.42
IL32	32	174	644
FAM231D	5.83	35.62	35.04
HES1	1493	6397	9470
IGFBP3	182	617	1455
PTX3	140	504	998
G0S2	602	2310	3421
CFAP57	42.9	127.07	314.12
NR4A2	57	144	411.03
GAGE12J	12.63	64.59	46.04
CXCL2	1207.98	2774.54	7900.02
DOK3	23	91	83
NTSR1	34	90	181
BIRC3	1678.03	6703	5701
SERTAD4	66	181	263
SLCO1B3	34	91	139
C15orf48	164.19	511	513
RELB	742	2130	2383
CLDN1	210	405	963
NAV3	150	295	659
HBEGF	72	194	230
OSGIN1	131	297	494
TNFRSF10D	240	706.01	645.99
PLAU	1131.91	2744.77	3683.91
SGK1	741	1723	2391
NFKB2	1180	3307	3032
MOK	340.01	698	1175.85
TRAF1	137	322	391
EBNA1BP2	5804.1	11879.93	18788.88

LOC400927-CSNK1E	54.58	143.56	129.13
ETS1	2174.04	4637.37	6119
KLF2	182	496	390
DNAJB6	3404	7214	9177
THSD1	135.28	353.72	286.02
LAMB3	238	465	654
SERPINE1	9897.11	19839.77	24798.97
SALL3	508	1160	1064
SNAPC1	1762.06	3395.31	4168.88
TBC1D2	278	534	619
SMURF2	2536.34	4870.63	5609.68
TAF4B	385	800	785
TMED7-TICAM2	239.78	461.11	488.68

Supplementary Materials and Methods

Cell lines

A total of six human lung cancer cell lines (H1299, H23, CL1-0, H1437, H358, and A549) and one immortalized lung cell line (BEAS-2B) were used in this study. They were obtained from Professor Yi-Ching Wang (National Cheng Kung University, Taiwan). Cells were cultured in Roswell Park Memorial Institute (*RPMI*) 1640 medium (GIBCO, Gaithersburg, MD, USA) supplemented with 2 mM L-glutamine, 1% nonessential amino acids (NEAA), 10% heat-inactivated fetal bovine serum (FBS), 100 U/ml penicillin and 100 μg/ml streptomycin (GE Healthcare Life Sciences, Chicago, IL, United States). All cell cultures were grown as monolayer cultures and maintained in a humidified atmosphere containing 5% CO_2 in air at 37°C.

DNA methylation

Genomic DNA was extracted from cell lines and tissue samples by using a commercial DNA extraction kit (QIAmp Tissue Kit; Qiagen, Hilden, Germany). We treated DNA with bisulfite using an EZ DNA methylation kit (Zymo Research, Orange, CA) as described previously. Briefly, 1 μg of genomic DNA was denatured by incubating with 0.2 M NaOH (Dilution Buffer). Aliquots of 10 mM hydroquinone and 3 M sodium bisulfite (pH 5.0) (CT Conversion Reagent) were added, and the solution was incubated at 50°C for 16 hr. Treated DNA was purified on a Zymo-Spin I column (Zymo Research), desulfonated with 0.3 M NaOH (Desulphonation Reagent), repurified on a Zymo-Spin I column and resuspended in 10μl elution buffer, and 60μl TE buffer. Following bisulfite treatment, all DNA samples were stored at −80°C [1-3]. CpG-methylated human genomic DNA (Thermo Fisher Scientific, San Diego, USA) and DNA extracted from normal peripheral blood lymphocytes were modified by sodium bisulfite to generate positive and negative controls, respectively. MSP, bisulfate sequencing and Q-MSP were performed as previously described [1,3,4]. For Q-MSP, the DNA methylation levels were assessed by determining the methylation index (MI) using the following formula: $100 \times 2^{-[(\text{Cp of Gene}) - (\text{Cp of COL2A})]}$.

Gene expression analysis

RNA extraction and reverse transcription polymerase chain reaction (RT-PCR) were conducted as previously described[1,3,4]. The primer sequences were described previously[3-6] and are shown in supplementary Table S1. We

isolated total RNA from each sample using the Qiagen RNeasy kit (Qiagen, Valencia, CA). One microgram of total RNA from each sample was subjected to reverse transcription using Superscript II reverse transcriptase and random hexamers (Invitrogen, Carlsbad, CA). Quantitative RT-PCR analysis was performed on an ABI 7500 Real-Time System (Applied Biosystems, Foster City, CA). Glyceraldehyde-3-phosphate dehydrogenase (*GAPDH*) was used as an internal control. PCRs were carried out using SYBR Green PCR Master Mix Reagents Kit. The mRNA levels of the interest genes were expressed as the ratio of the interest gene to *GAPDH* mRNA, for each sample.

Western blot analysis.

Cells were scraped from the dish and lysed for 30 min in lysis buffer. Concentration of protein was determined using the BCA kit (Pierce, Rockford, IL, CA). Total cellular proteins (50 μg per lane) were separated by SDS-PAGE and then transferred to PVDF membranes. After blocking, membranes were then incubated with specific antibody. After washing with blocking buffer, membranes were incubated with horseradish peroxidase-conjugated goat anti-rabbit (1:1000) or goat anti-mouse (1:5000) IgG. Specific protein bands were developed using the Amersham ECL nonradioactive method (Amersham, Piscataway, NJ).

Plasmids and shRNA clones

SOX1 full length open-reading frame (ORF) cDNA product was constructed into pcDNA3.1-V5-His-TOPO constitutive expression vector (Invitrogen, USA) (termed pcDNA3.1-SOX1) or the inducible expression vector pT-Rex-DEST3.1 (termed pT-Rex-DEST3.1-SOX1), respectively. The pLKO.1-shLacZ and HES1-shRNA were purchased from National RNAi Core Facility of Taiwan. The pENTER-HES1 plasmid was purchased from biotech company (ViGene BioSciences). The shRNA sequences were described in Supplementary Table S1.

Transfection

Cells plated in 100 mm dishes were transfected at 50–80% confluence with *SOX1* expression vectors or with control vectors, using the liposome-mediated transfection method. To establish cells stably expressing *SOX1,* H23 and H1299 cells were transfected with the plasmid of choice (pcDNA3.1-SOX1 or pT-REx-

DEST-SOX1) for two days, then trypsinized and plated at low density. Stable mixed clones were selected by maintaining cells in medium containing the G418 or blasticidin antibiotic.

Tet on inducible expression system

To build up an inducible system, pcDNA6/TR (containing the tetracycline repressor gene) stable clones were selected and then transfected with pT-REx-DEST-SOX1vector to generate cells expressing SOX1 under doxycycline (DOX) (Invitrogen) induction. Two days after transfection, 0.9 mg/ml G418 solution and 7.5 μg/ml blasticidin (Invitrogen) were added to cells for selection of stable clones as mentioned before [3]. To validate the inducible stable clones, SOX1 expression was induced for 48 hr with 1 μg/ml DOX prior to harvesting for Western blot. Stable clones were selected and maintained in medium containing 0.3 mg/ml G418 w/o 2.5 μg/ml blasticidin antibiotics.

MTS Proliferation Assay.

Cells (3×10^3) were seeded in 96-well plates and allowed to adhere overnight. MTS assays were performed with the Cell Titer 96 AQ One Solution Cell Proliferation Assay (Promega Corporation, Madison, WI). MTS reagent (20 μl/well) was added to 100 μl of medium containing cells in each well of 96-well plates and left for 4 h at 37 °C under humidified 5% CO_2 in air. For colorimetric analysis, the absorbance at 490 nm was recorded using a microplate reader. Each condition was repeated at least three times. Total cells were harvested at the designated times after treatment.

Colony formation.

Cells were trypsinized and resuspended in 1.5 ml of 0.5% agarose, then poured onto a 1.5 ml 1% agarose bed in 35 mm tissue culture dishes. After 3–4 weeks, cells were stained with a solution containing 0.005% crystal violet, 1.9% formaldehyde and 0.15 M NaCl, for 30 min. After washing and drying, colonies larger than 1 mm were counted.

Invasion assay.

In vitro cell invasion was determined in 24-well Transwell plates (Coster) with Matrigel (BD Biosciences) coating. Stable transfectants expressing SOX1 were suspended in optimum medium at a concentration of 1~ 5 x 10^4 cells/ml, placed in the upper chambers, and incubated for 16–24 h at 37 °C in 5% CO_2. Noninvasive cells in the upper chamber were subsequently removed with a cotton-tip applicator. Invasive cells on the lower surface were fixed with methanol and stained with hematoxylin. The number of invasive cells were determined by counting 10 high-power fields (100× magnification) on each membrane and calculated as the mean number of cells per field. All cell lines were assayed in duplicate for each experiment, and each experiment was repeated three times.

Chromatin immunoprecipitation assay (ChIP assay)

Cells were fixed with 1% formaldehyde and then quenched with glycine, followed by preparation of nuclear lysates using the EZ-Magna ChIP G Kit (Millipore, Billerica, MA, USA). ChIP assays were performed using an according to the manufacturer's protocol. Briefly, Nuclear lysates were sonicated to obtain DNA fragments around 500 bp and then subjected to immunoprecipitation for 16 h at 4 °C using 4 μg anti-SOX1 antibody (R&D Systems, Minneapolis, MN, USA). An antibody against mouse IgG (Millipore, USA) was used as a negative control, and an anti-H3K27me3 antibody (Millipore, USA) was used to evaluate the degree of chromatin transcriptional level. The IPs and input DNA were purified and quantified by quantitative PCR using SOX1 binding site-specific primers.

1. Lai, H.C.; Lin, Y.W.; Huang, T.H.; Yan, P.; Huang, R.L.; Wang, H.C.; Liu, J.; Chan, M.W.; Chu, T.Y.; Sun, C.A., et al. Identification of novel DNA methylation markers in cervical cancer. *Int J Cancer* **2008**, *123*, 161-167, doi:10.1002/ijc.23519.
2. Lin, Y.W.; Tsao, C.M.; Yu, P.N.; Shih, Y.L.; Lin, C.H.; Yan, M.D. SOX1 suppresses cell growth and invasion in cervical cancer. *Gynecologic oncology* **2013**, *131*, 174-181, doi:10.1016/j.ygyno.2013.07.111.
3. Tsao, C.M.; Yan, M.D.; Shih, Y.L.; Yu, P.N.; Kuo, C.C.; Lin, W.C.; Li, H.J.; Lin, Y.W. SOX1 functions as a tumor suppressor by antagonizing the

WNT/beta-catenin signaling pathway in hepatocellular carcinoma. *Hepatology* **2012**, *56*, 2277-2287, doi:10.1002/hep.25933.
4. Liu, C.Y.; Chao, T.K.; Su, P.H.; Lee, H.Y.; Shih, Y.L.; Su, H.Y.; Chu, T.Y.; Yu, M.H.; Lin, Y.W.; Lai, H.C. Characterization of LMX-1A as a metastasis suppressor in cervical cancer. *J Pathol* **2009**, *219*, 222-231, doi:10.1002/path.2589.
5. Chung, H.H.; Lee, C.T.; Hu, J.M.; Chou, Y.C.; Lin, Y.W.; Shih, Y.L. NKX6.1 Represses Tumorigenesis, Metastasis, and Chemoresistance in Colorectal Cancer. *International journal of molecular sciences* **2020**, *21*, doi:10.3390/ijms21145106.
6. Li, H.J.; Yu, P.N.; Huang, K.Y.; Su, H.Y.; Hsiao, T.H.; Chang, C.P.; Yu, M.H.; Lin, Y.W. NKX6.1 functions as a metastatic suppressor through epigenetic regulation of the epithelial-mesenchymal transition. *Oncogene* **2016**, *35*, 2266-2278, doi:10.1038/onc.2015.289.
